# An assessment of burden associated with problem joints in children and adults with moderate or severe haemophilia A: analysis of the CHESS-Paediatrics and CHESS II cross-sectional studies

**DOI:** 10.1186/s13023-024-03514-1

**Published:** 2025-01-13

**Authors:** Paul McLaughlin, Hortensia De la Corte-Rodriguez, Tom Burke, Francis Nissen, Martynas Aizenas, Katya Moreno, Jamie O’Hara

**Affiliations:** 1 The Katharine Dormandy Haemophilia Centre and Thrombosis Unit, Royal Free London NHS Foundation Trust, London, UK; 2 Department of Physical and Rehabilitation Medicine, La Paz University Hospital (IdiPaz), Madrid, Spain; 3 HCD Economics, Mere House, Brook St, Knutsford, Cheshire, WA16 8GP UK; 4 University of Chester, Chester, Cheshire UK; 5 F. Hoffmann-La Roche Ltd, Basel, Switzerland

**Keywords:** Haemophilia A, Factor VIII, Problem joint, Adherence, Physical activity, Healthcare resource use, Burden

## Abstract

**Background:**

Clinical research has offered many definitions and fragmented perspectives of joint morbidity in haemophilia. As joint damage, pain and mobility impairment can be present without clinical record of persistent bleeding, a person-centric joint morbidity characterisation remained a priority for the haemophilia community, giving rise to the ‘problem joint’ concept.

As diagnosing and managing joint morbidity is critical, the aim of this study was to analyse the holistic burden of problem joints in people with moderate or severe haemophilia A (HA).

Data from the ‘Cost of Haemophilia in Europe: a Socioeconomic Survey’ (CHESS) cross-sectional studies were used. CHESS-Paediatrics included male paediatric patients (≤ 17 years) with congenital moderate or severe haemophilia, while CHESS II included adult males (≥ 18 years) of any severity. Both studies sought to collect detailed information on the clinical, economic and humanistic burden of haemophilia.

Demographics, clinical outcomes, treatment regimen, adherence, physical activity, healthcare resource use and number of problem joints were evaluated and described by HA severity and number of problem joints (none, 1, ≥ 2).

**Results:**

In total, 1171 people with non-inhibitor moderate or severe HA from CHESS-Paediatrics (*n* = 703) and CHESS II (*n* = 468) were included in this analysis.

Presence of problem joints was more prevalent among CHESS II participants (44%) than in CHESS-Paediatrics (14%). Around two-thirds (67%) of CHESS-Paediatrics and 39% of CHESS II participants received prophylactic factor VIII replacement therapy. The presence of chronic pain was greater in severe HA with ‘ ≥ 2’ problem joints in both cohorts. Clinical symptoms and bleed-related hospitalizations were more prevalent in the presence of problem joints regardless of HA severity in both cohorts.

**Conclusions:**

This analysis of the CHESS population studies has expanded on previous work by examining the relevance of the problem joint measure of haemophilic morbidity and its associated burden. Adverse clinical symptoms and increased bleed-related hospitalizations were observed in the presence of problem joints in both children/adolescents and adults across HA severities. Use of person-centric characterizations of joint morbidity may improve analysis of long-term outcomes and lead to improvements in future haemophilia care.

## Background

Haemophilia A (HA) is a rare genetic disorder defined by a lack of endogenous clotting factor VIII. HA occurs predominantly in males, with a worldwide prevalence of 17.1 per 100,000 males overall [1]. The level of factor VIII determines the severity, where people with mild, moderate, or severe HA have > 5 to 40 IU/dL, 1–5 IU/dL, and < 1 IU/dL, respectively; severe HA prevalence is approximately 6 cases per 100,000 males [1, 2]. Spontaneous bleeding into joints is of particular concern for people with moderate or severe HA, as, without appropriate treatment, 9 out of 10 people with severe haemophilia are likely to have chronic degenerative changes in up to 6 major joints by their second or third decade of life [3]. This long-term joint deterioration reduces quality of life and daily functioning while increasing the need for related healthcare services [4–7].

Historically, clinical research and practice have utilized numerous definitions of haemophilic joint morbidity, offering a fragmented perspective on joint morbidity over time and across care settings [2, 8–11]. The most recent and common definition of a ‘target joint’ is determined by the recording of ≥ 3 spontaneous bleeds in the same joint over a continuous 6-month period [2]. As underlying joint damage, pain, and mobility impairment can all be present without clinical recording of persistent bleeds, these target joint definitions are likely to lead to underdiagnosis of joints otherwise worthy of clinical attention. As such, a person-centric characterization of joint morbidity has remained a priority for the haemophilia community, and gave rise to the formulation of the ‘problem joint’ concept [12, 13]. A problem joint is defined by presence of chronic joint pain and/or limited range of movement due to compromised joint integrity (i.e., chronic synovitis and/or haemophilic arthropathy), with or without persistent bleeding [12, 13].

Persistent joint bleeds are known to cause substantial long-term burden to people with moderate or severe HA, and a factor VIII prophylaxis regimen early in life has been shown to improve joint-related outcomes through adolescence [14]. While the target joint concept has historically provided meaningful clinical utility and consistency in research, our understanding of haemophilic joint morbidity through the lens of the person-centred ‘problem joint’ definition is lacking. Given the importance of diagnosing and managing joint morbidity through the lifespan, we conducted an analysis on a subset of adult and paediatric patients with the aim of analysing the holistic burden of problem joints. The analysis was conducted in those with moderate or severe haemophilia A, without diagnosis of inhibitors, who participated in the Cost of Haemophilia in Europe: a Socioeconomic Survey II (CHESS II) and Cost of Haemophilia in Europe: a Socioeconomic Survey Paediatrics (CHESS Paediatrics) cross-sectional retrospective studies.

## Results

### Participants and problem joints

A subset of 1,171 people with moderate or severe HA and no inhibitors from CHESS Paediatrics (*n* = 703) and CHESS II (*n* = 468) who met the inclusion criteria were included from CHESS Paediatrics (*n* = 992) and CHESS II (*n* = 787). The paediatric cohort (mean age, 10.3 years) was evenly distributed across participating European countries, while most of the adult cohort (mean age, 38.7 years) were recorded in Italy or Spain (72%, 337/468; Table 1). Similar proportions of each cohort had severe HA (59% in each; CHESS Paediatrics, 416/703; CHESS II, 278/468).

**Table 1:** Participant characteristics by HA severity and number of problem joints

Study, characteristic	Total	Moderate HA			Severe HA		
		0 PJ	1 PJ	≥ 2 PJs	0 PJ	1 PJ	≥ 2 PJs
CHESS Paediatrics, n (%)	703 (100)	254 (89)	22 (8)	11 (4)	350 (84)	52 (13)	14 (3)
Age, mean (SD), years	10.3 (4.70)	10.5 (4.68)	13.0 (3.63)	9.9 (4.64)	9.9 (4.73)	10.5 (4.74)	12.4 (4.4)
Weight, mean (SD), kg	41.6 (18.68)	42.1 (19.28)	49.9 (15.39)	38.6 (17.74)	40.3 (18.41)	42.5 (17.58)	50.1 (20.14)
BMI, mean (SD), kg/m^2^	21.2 (4.10)	21.0 (3.69)	21.8 (5.08)	23.1 (6.86)	20.9 (4.10)	22.9 (4.46)	22.9 (3.60)
Country, n (%) Spain Italy France Germany The United Kingdom	168 (24) 167 (24) 148 (21) 112 (16) 108 (15)	84 (33) 80 (32) 22 (9) 37 (15) 31 (12)	10 (46) 2 (9) 0 6 (27) 4 (18)	9 (82) 0 0 1 (9) 1 (9)	46 (13) 75 (21) 113 (32) 58 (17) 58 (17)	17 (33) 7 (14) 7 (14) 8 (15) 13 (25)	2 (14) 3 (21) 6 (43) 2 (14) 1 (7)
Number of comorbidities, n (%) 0 1 2 ≥ 3	550 (78) 95 (14) 32 (5) 26 (4)	200 (79) 33 (13) 15 (6) 6 (2)	16 (73) 2 (9) 2 (9) 2 (9)	4 (36) 4 (36) 2 (18) 1 (9)	294 (84) 42 (12) 7 (2) 7 (2)	27 (52) 10 (19) 6 (12) 9 (17)	9 (64) 4 (29) 0 1 (7)
Top comorbidities, n (%) Gingivitis Anxiety Anaemia ADHD Osteoarthritis	70 (10) 56 (8) 50 (7) 38 (5) 26 (4)	24 (9) 23 (8) 26 (9) 18 (6) 7 (2)			46 (11) 33 (8) 24 (6) 20 (5) 19 (5)		
CHESS II, n (%)	468 (100)	116 (61)	44 (23)	30 (16)	147 (53)	73 (26)	58 (21)
Age, mean (SD), years	38.7 (13.95)	37.3 (13.60)	39.2 (13.86)	50.4 (16.2)	36.5 (12.11)	37.3 (13.9)	42.4 (14.8)
Weight, mean (SD), kg	74.5 (10.39)	75.8 (10.8)	72.6 (11.1)	76.7 (9.01)	72.9 (9.61)	76.34 (9.47)	73.7 (12.10)
BMI, mean (SD), kg/m^2^	24.6 (3.06)	24.4 (2.63)	25.0 (2.73)	24.2 (2.77)	25.5 (3.12)	24.5 (2.70)	24.6 (2.90)
Country, n (%) Italy Spain The United Kingdom France Germany Romania The Netherlands	188 (40) 149 (32) 48 (10) 42 (9) 37 (8) 3 (1) 1 (0.2)	36 (31) 34 (29) 15 (13) 16 (14) 13 (11) 2 (2) 0	24 (55) 14 (32) 3 (7) 0 3 (7) 0 0	13 (43) 16 (53) 0 0 0 0 1 (3)	44 (30) 47 (32) 18 (12) 22 (15) 15 (10) 1 (1) 0	34 (47) 20 (27) 12 (16) 3 (4) 4 (6) 0 0	37 (64) 18 (31) 0 1 (2) 2 (3) 0 0
Number of comorbidities, n (%) 0 1 2 ≥ 3	223 (48) 98 (21) 72 (15) 75 (16)	70 (60) 23 (20) 13 (11) 10 (9)	14 (32) 7 (16) 13 (30) 10 (23)	4 (13) 7 (23) 9 (30) 10 (33)	89 (61) 32 (22) 16 (11) 10 (7)	31 (42) 17 (23) 10 (14) 15 (21)	15 (26) 12 (21) 11 (19) 20 (34)
Top comorbidities, n (%) Anxiety Smoking Depression Fatigue Anaemia	86 (18) 77 (16) 49 (10) 47 (10) 45 (10)	36 (19) 30 (16) 23 (12) 23 (12) 17 (9)			50 (18) 47 (17) 28 (10) 26 (9) 24 (9)		

ADHD, attention deficit-hyperactivity disorder; HA, haemophilia A; PJ, problem joints; SD, standard deviation

**Fig. 1:**
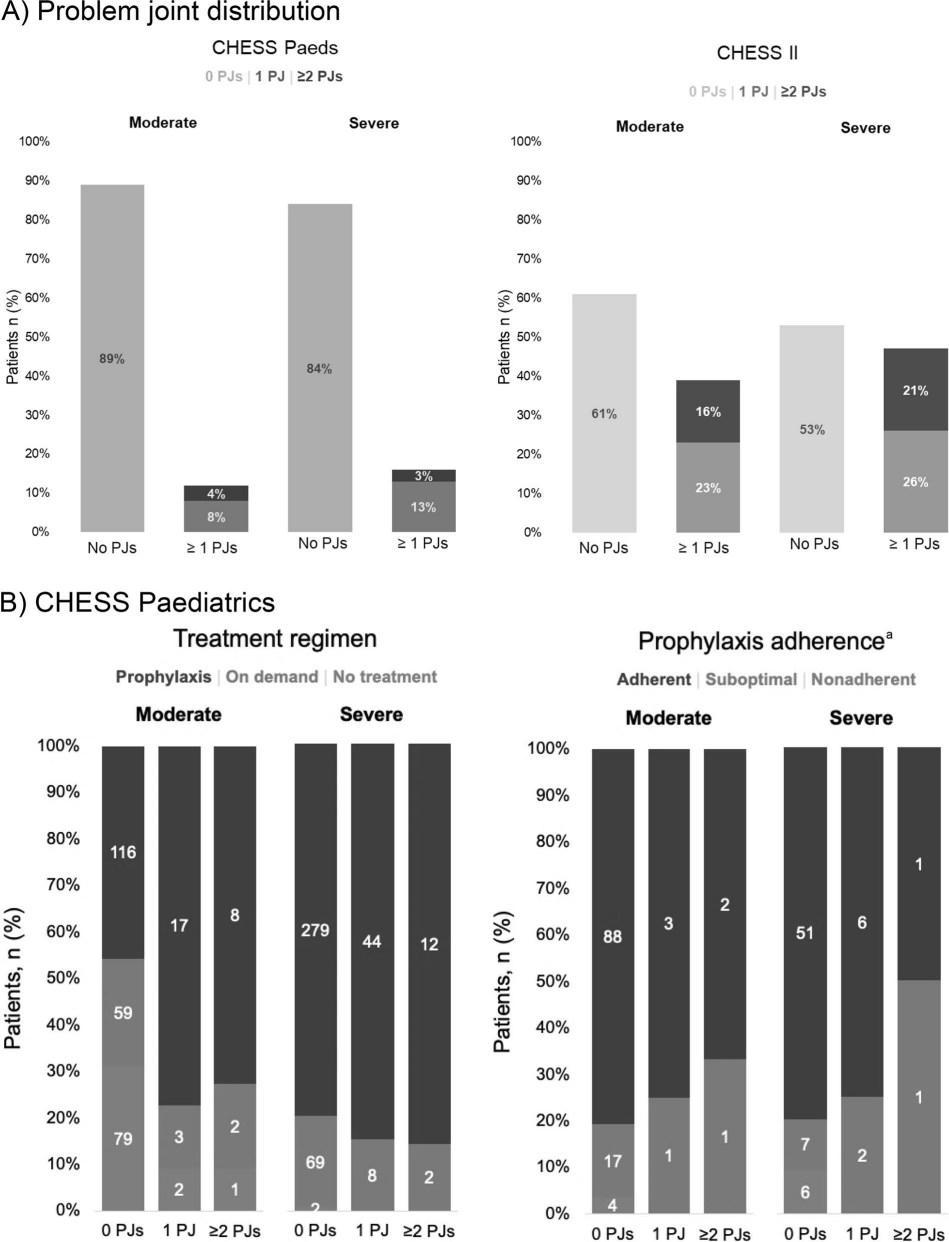

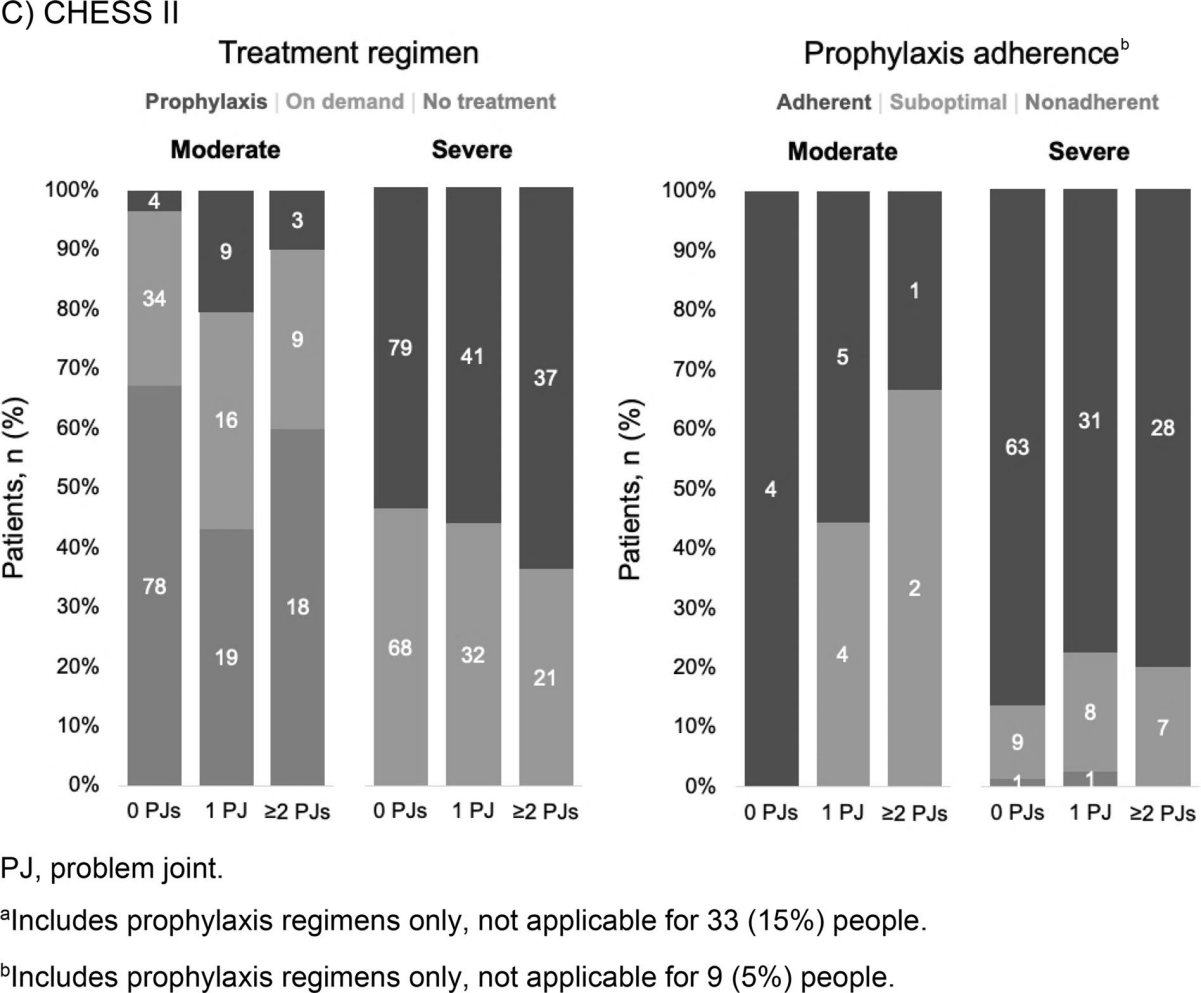
Problem Joints, Treatment regimens and adherence. **A** Problem joint distribution. **B** CHESS Paediatrics. **C** CHESS II. PJ, problem joint. ^a^Includes prophylaxis regimens only, not applicable for 33 (15%) people. ^b^Includes prophylaxis regimens only, not applicable for 9 (5%) people

Overall, having ≥ 1 problem joint was more prevalent among adults in CHESS II (44%) than children and adolescents in CHESS Paediatrics (14%; Table 1, Fig. 1). In the paediatrics cohort, 11% of those with moderate HA (*n* = 33/287) and 16% with severe HA (66/416) had ≥ 1 problem joint. Participants had a mean (SD) of 0.20 (0.50) problem joints (mean of 0.14 and 0.24 in moderate and severe HA, respectively). These proportions were higher in adults captured in CHESS II, both for those with moderate HA (39%, 74/190) and severe HA (47%, 131/278). Participants had a mean (SD) of 0.58 (0.93) problem joints (mean of 0.35 and 0.74 with moderate and severe HA, respectively).

### Treatment, physical activity and clinical outcomes

**Fig. 2:**
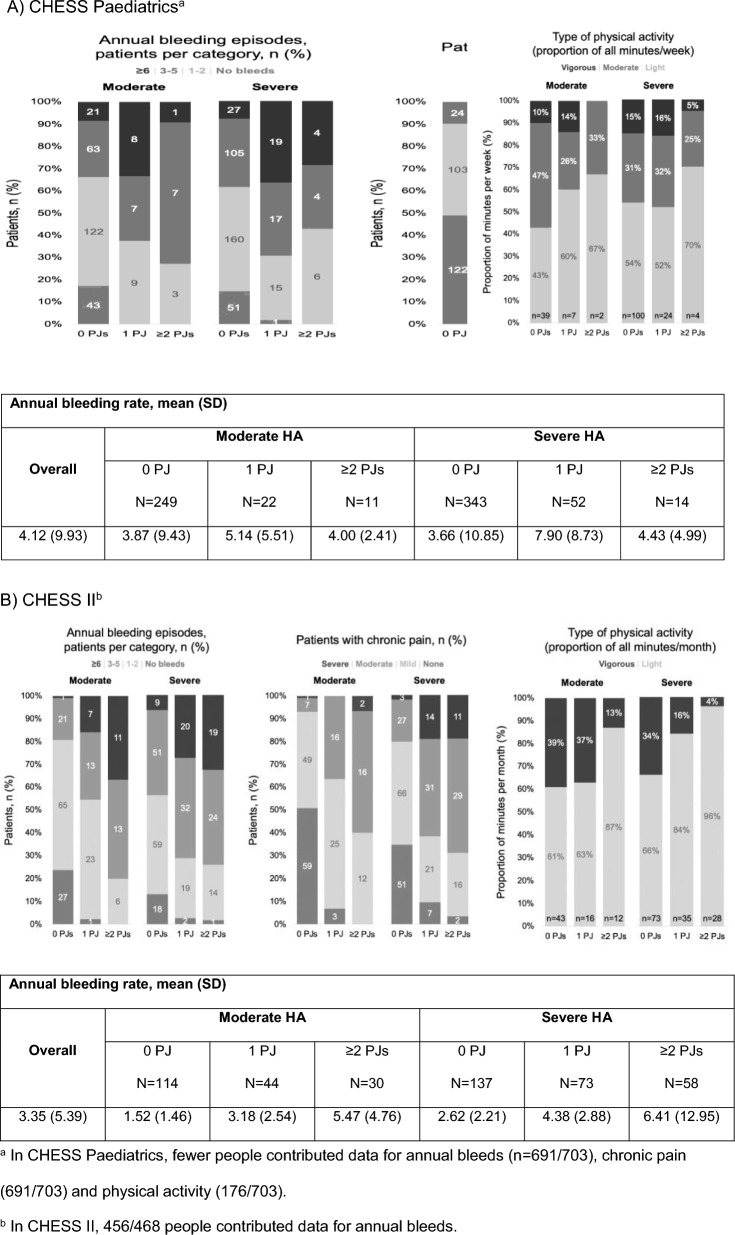
Bleeding events, chronic pain and physical activity. **A** CHESS Paediatrics^a^. **B** CHESS II^b^. ^a^In CHESS Paediatrics, fewer people contributed data for annual bleeds (n = 691/703), chronic pain (691/703) and physical activity (176/703).^b^ In CHESS II, 456/468 people contributed data for annual bleeds

The majority of paediatrics participants received a prophylaxis regimen (67%). Of those, 45.6% received primary prophylaxis (prophylaxis initiated before the age of 2 and before the occurrence of more than one joint bleed), 19.2% received secondary prophylaxis (prophylaxis started after the age of two and/or after one or more joint bleeds) and 2.8% received tertiary prophylaxis (prophylaxis initiated after the onset of joint damage, irrespective of age). slightly more than one-third (37%) of adult participants were receiving prophylaxis (Fig. 1) Of those receiving prophylaxis, the majority were receiving secondary prophylaxis (74.6%) and some receiving tertiary prophylaxis (23.7%), with a very small minority on primary prophylaxis (1.7%). Overall treatment adherence was generally high in both paediatrics and adults (Fig. 1). The presence of chronic pain was greater in severe HA in those with ‘ ≥ 2’ problem joints. Nearly all paediatrics participants with severe HA and ‘ ≥ 2’ problem joints reported mild or moderate chronic pain (13/14; 93%). The majority of adult participants with severe HA and ‘ ≥ 2’ problem joints reported moderate or severe chronic pain (40/58, 69%; Fig. 2). The proportion of adult participants to experience bleeding episodes in the previous 12 months increased with number of problem joints (Fig. 2). Vigorous physical activity accounted for only 26–27% of all activity measured across groups in paediatrics and adults, respectively, and reported a smaller degree of physical activity with increasing number of problem joints (Fig. 2).

### Healthcare resource use

**Fig. 3:**
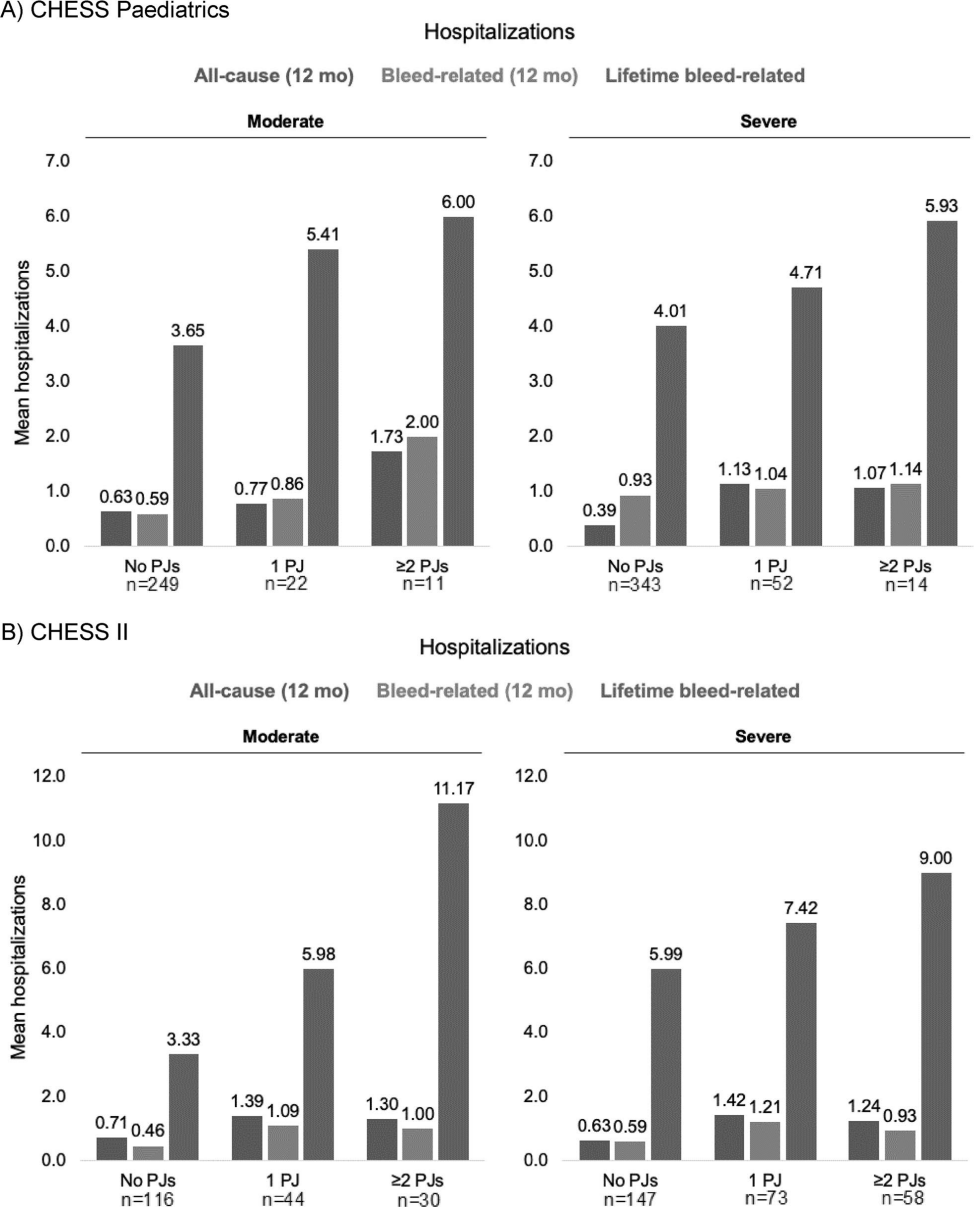
All-cause and bleed-related hospitalizations. **A** CHESS Paediatric. **B** CHESS II

‘All-cause’ and ‘bleed-related’ hospitalizations in the paediatric cohort appeared higher among those with more problem joints, across severity groups. Those with severe HA in the ‘none’ versus ‘ ≥ 2’ problem joint groups had mean ‘all-cause’ hospitalizations in the past 12 months of 0.39 (SD, 1.06) and 1.07 (2.64), respectively, and mean ‘bleed-related’ hospitalizations of 0.93 (7.30) and 1.14 (2.63). In adults with severe HA, mean ‘all-cause’ hospitalizations among those with ‘none’ versus ‘ ≥ 2’ problem joints were 0.63 (1.00) and 1.24 (1.25), and mean bleed-related hospitalizations were 0.59 (0.97) and 0.92 (1.02; Fig. 3).

## Discussion

This retrospective analysis of the cross-sectional CHESS Paediatrics and CHESS II studies found that presence and number of problem joints was associated with clinical burden with in people with moderate or severe HA, as well as associated increases in healthcare resource utilization. Despite the prevalence of prophylaxis factor replacement therapy, problem joints were present in a substantial proportion of both cohorts. Among paediatric patients, 12–16% of the moderate and severe cohorts respectively were reported to suffer from at least one problem joint. On the other hand, in the adult cohort, 39–47% of moderate and severe patients respectively had at least one problem joint. Clinical outcomes and physical activity levels worsened in the presence of problem joints, regardless of HA severity. Similarly, ‘all-cause’ and ‘bleed-related’ hospitalizations in the previous 12 months increased with the presence of problem joints, as did lifetime ‘bleed-related’ hospitalizations.

Early-life initiation of prophylactic treatment is the recommended treatment approach in patients with severe HA or severe bleeding phenotype, as it has been shown to improve longer-term outcomes among children and adolescents with severe HA, emphasizing the connection between joint health and clinical outcomes [13–17]. Generally, high rates of prophylaxis treatment were observed in the CHESS Paediatrics population (46–73% in moderate HA; 80–86% in severe HA). Nonetheless, problem joints were observed in 14% (99/703) of the overall paediatric cohort. Prophylaxis treatment regimens were less common among adults (3–21% in moderate HA; 54–64% in severe HA), and the prevalence of problem joints across the cohort was high (44%; 205/468). The observed prevalence of problem joints was similar among participants with moderate or severe HA in both paediatric and adult populations, potentially highlighting the need for further research and access to a high standard of treatment in people with moderate disease. The evaluation of clinical outcomes of participants of this study may offer new insights into the real-world burden of joint morbidity throughout the lifespan of people with haemophilia. With respect to the problem joint outcome measure, the prevalence of the problem joints aligned with reported chronic pain, physical activity, annualized bleeding events and patient-reported instruments. Other published analysis of these data has shown worsening quality of life (EQ-5D scores) and increased direct and indirect costs in the presence of problem joints [18, 19]. This study found lower levels of vigorous activity among adults with ‘ ≥ 2’ problem joints regardless of severity, as well as a smaller overall proportion of people reporting vigorous versus light activity with severe HA (4% with ‘ ≥ 2’ problem joints versus 34% with ‘none’) when compared with moderate HA (13% with ‘ ≥ 2’ problem joints versus 39% with ‘none’). Previous research into work and activity impairment in the CHESS II cohort has found the greatest decrease in productivity levels was between those with ‘ ≥ 2’ problem joints vs. ‘none’ for moderate HA, and between ‘none’ and ‘1’ problem joint with severe HA, when comparing joint damage in ‘none’, ‘1’, and ‘ ≥ 2’ problem joints, as reported in this analysis. Absenteeism was reported among 1% of adult participants versus 10% of those with moderate HA and ‘none’ vs. ‘ ≥ 2’ problem joints, respectively, and 8% versus 12% in those with severe HA and ‘none’ vs. ‘1’ problem joint (7% for ‘ ≥ 2’ problem joints) [18, 19]. Taken together, these findings show a worsening holistic burden in the presence of one or more problem joints, as the chronic pain, reduced physical activity, bleeding events, and associated hospitalizations observed in this analysis are broadly consistent with the reduced productivity and increased costs observed in the complementary analysis. These findings may underline the importance of detecting problem joints in order to offer treatment and lifestyle strategies that can help to improve the effectiveness treatment, adherence, functionality, and pain management, with the aim of reducing the impact on the lives and well-being of people with HA and their families.

These findings were based on cross-sectional observational data and, as such, are subject to certain inherent limitations related to the secondary nature of the analysis, as well as potential for information and/or recall bias. Participation in the study was entirely voluntary and inclusion of clinical data in the study was contingent on individuals having at least one consultation within the 12 months prior to data collection. Therefore, despite efforts to minimise it, a degree of selection bias and potential underrepresentation of certain patient categories that may consult less frequently cannot be excluded [20, 21]. Additionally, it is important to consider the variability and heterogeneity of joint health assessment across haemophilia care practices [22] and how these may impact results: as this cross-sectional study was based on information available in patient charts at the time of data collection (to avoid any impact or modification of routine clinical care), the protocol did not prescribe a specific method of joint assessment. Joint health information was collected via the Problem Joint tool, developed to capture holistic joint impact even in the absence of formal recorded joint health assessments [13].

However, participant-reported outcomes are essential to appropriately managing this lifelong, symptomatic bleeding disorder, particularly among those with moderate or severe disease, and real-world data is important to understand physician awareness of symptoms and related outcomes. It may be important for clinicians to use methods that are sensitive to early detection of joint pain and damage before people with HA suffer further pain and limited range of motion [23]. This analysis utilised small sample sizes in some subgroups of people with HA and all were treated with FVIII concentrates. Additionally, in the CHESS II dataset, a larger representation of patients from Spain and Italy was observed, therefore findings should be interpreted with care, as this may limit the generalisability to the European population. Future studies evaluating larger subgroups of those identified here with the greatest prevalence and burden of problem joints, and including non-factor therapies, are warranted and would be valuable additions to this work in the context of the evolving treatment landscape.

## Conclusion

This analysis of the CHESS population studies has extended previous work illustrating the relevance of the problem joint measure of haemophilic arthropathy and its associated burden on people with haemophilia. Worsening clinical symptoms and increased bleed-related hospitalizations were observed in the presence of problem joints regardless of HA severity in both children/adolescents and adults. Greater attention to person-centric characterizations of joint morbidity may improve the use of more effective treatment regimens, adherence, and holistic long-term outcomes.

## Methods

### The CHESS-Paediatrics and CHESS II studies

The current analysis utilized data drawn from the retrospective cross-sectional ‘Cost of Haemophilia in Europe: a Socio-economic Survey II’ (CHESS II) and Cost of Haemophilia in Europe: a Paediatric Socio-economic survey CHESS Paediatrics studies. The study design and preliminary findings of the CHESS study (severe only, carried out in 2016) [24], as well as for the CHESS-Paediatrics study in children and CHESS II study in European adults have been reported previously [20, 25–29]. Both CHESS II and CHESS Paediatrics are panel-based cross-sectional burden of illness studies of people with congenital haemophilia A or B, with or without inhibitors. The inclusion criteria for the paediatric study were that all participants had to be male children or adolescents (≤ 17 years old), with moderate or severe congenital haemophilia, living in France, Germany, Italy, Spain, and the United Kingdom at the time of data collection (2017–2018). Caregivers provided information for their children (< 8 years old) with haemophilia. Similarly, inclusion criteria for the CHESS II study were that participants had to be adult males (≥ 18 years) with congenital haemophilia of any severity, living in Germany, Spain, France, Italy, Romania, The Netherlands, Denmark and the United Kingdom at the time of data collection (2018–2019). Haemophilia healthcare practitioners completed a web-based clinical record form reporting on demographic, clinical characteristics, treatment patterns and health-resource utilisation outcomes abstracted from medical notes. Patients (or caregivers/guardians if patients were < 18) were invited by the healthcare practitioner to complete a patient questionnaire, and upon providing informed consent provided information on non-medical haemophilia related cost, their health status, productivity and labour market outcomes as well as information on the overall socioeconomic and humanistic impact of haemophilia. In both studies, to minimise selection bias, the physicians were instructed to recruit the next eight eligible patients with whom they consulted, irrespective of the reason of the consultation (routine or emergency).

Both the CHESS II and CHESS Paediatrics studies were approved by the Research Ethics Sub-committee of the Faculty of Health and Social Care within the university of Chester, conducted in correspondence with regional and relevant guidelines and overseen by a steering committee composed by clinical haemophilia experts, patient representatives and patient advocacy organisation members from the countries involved. The research presented was intended to focus on the burden of moderate and severe HA, and therefore people with mild HA (CHESS II, *n* = 100), haemophilia B (CHESS II, *n* = 159; CHESS Paediatrics, *n* = 206) or a diagnosis of inhibitor (CHESS II, *n* = 27;CHESS Paediatrics, *n* = 82) were excluded. CHESS II (*n* = 33) participants aged 18–19 years old were also excluded from the present analysis in order to avoid potential overlapping participation across studies (in cases CHESS-Paediatrics participants aged 16–17 may have later enrolled in CHESS II, years later).

### Outcome measures

Participant demographics, clinical characteristics and outcomes, treatment regimen, adherence, physical activity, healthcare resource use (HRU), and number of problem joints were evaluated according to HA severity (moderate or severe). Demographic and clinical characteristics included age, weight, body mass index (BMI), country, and comorbidities. Problem joints were reported as continuous and categorical variables according to the published definition of a problem joint and based on physician-reported information abstracted from the medical charts [12, 30]. Direct and indirect costs have been analysed separately [18, 19].

Clinical outcomes included annual bleeding rate (including joint bleeds) and chronic pain (none, mild, moderate, severe) as reported by the physician. Treatment regimens included factor VIII regimen (prophylaxis [defined as “regular continuous treatment / preventative therapy received regularly”], on demand [defined as “treatment given at the time of clinically evident bleeding / in response to a bleed”], or none) and factor VIII consumption in the 12 months prior to index date (consultation at which participant was included in study). Adherence, as reported by the treating physician, was categorized as fully adherent (> 85% intended infusions), sub optimally adherent (75–85%), and non-adherent (< 75%) overall and among those receiving prophylaxis. HRU measures included ‘all-cause’ and ‘bleeding-related’ hospitalizations in the past 12 months, and lifetime ‘bleeding-related’ hospitalizations. All outcomes pertaining to demographic and clinical characteristics, as well as information on clinical outcomes, treatment regimens, adherence to treatment and HRU were reported by the physician.

Physical activity was self-reported as minutes-per-week and categorized by light, moderate, or vigorous activity in CHESS-Paediatrics, and as minutes-per-month and categorized by light or vigorous activity in CHESS II. For CHESS II, participants indicated which activities they performed more than 10 times in the past year, with the average number of times per month and average number of minutes for each session.

### Statistical analysis

Demographic and clinical characteristics, clinical outcomes and HRU measures were summarized descriptively by HA severity (moderate or severe) and number of problem joints (none, 1, ≥ 2). No imputation of missing values was performed. All analyses were conducted using STATA version 16.0 (www.stata.com).

## Data Availability

The data that support the findings of this study may be available from HCD Economics, Ltd but restrictions apply to the availability of these data, which were used under license for the current study, and so are not publicly available. Data may be available from the authors upon reasonable request and with permission of HCD Economics Ltd.

## List of Abbreviations

CHESS: Cost of Haemophilia in Europe: a Socioeconomic Survey
HA: Haemophilia A
SD: Standard Deviation
PJ: Problem joint
